# Influenza and other respiratory virus infections in outpatients with medically attended acute respiratory infection during the 2011-12 influenza season

**DOI:** 10.1111/irv.12247

**Published:** 2014-04-08

**Authors:** Richard K Zimmerman, Charles R Rinaldo, Mary Patricia Nowalk, Balasubramani GK, Mark G Thompson, Krissy K Moehling, Arlene Bullotta, Stephen Wisniewski

**Affiliations:** a Department of Family Medicine, University of Pittsburgh School of MedicinePittsburgh, PA, USA; b Department of Pathology, University of Pittsburgh School of MedicinePittsburgh, PA, USA; c Department of Infectious Disease and Microbiology, University of Pittsburgh Graduate School of Public HealthPittsburgh, PA, USA; d Department Epidemiology, University of Pittsburgh Graduate School of Public HealthPittsburgh, PA, USA; e Influenza Division, Centers for Disease Control and PreventionAtlanta, GA, USA

**Keywords:** Co-detection, influenza, RT-PCR, viral infections

## Abstract

**Background:**

Respiratory tract infections are a major cause of outpatient visits, yet only a portion is tested to determine the etiologic organism. Multiplex reverse transcriptase polymerase chain reaction (MRT-PCR) assays for detection of multiple viruses are being used increasingly in clinical settings.

**Methods:**

During January–April 2012, outpatients with acute respiratory illness (≤7 days) were tested for influenza using singleplex RT-PCR (SRT-PCR). A subset was assayed for 18 viruses using MRT-PCR to compare detection of influenza and examine the distribution of viruses and characteristics of patients using multinomial logistic regression.

**Results:**

Among 662 participants (6 months–82 years), detection of influenza was similar between the MRT-PCR and SRT-PCR (κ = 0·83). No virus was identified in 267 (40.3%) samples. Commonly detected viruses were human rhinovirus (HRV, 15·4%), coronavirus (CoV, 10·4%), respiratory syncytial virus (RSV, 8·4%), human metapneumovirus (hMPV, 8·3%), and influenza (6%). Co-detections were infrequent (6·9%) and most commonly occurred among those <18 years old. In regression analyses, compared with non-viral illnesses, RSV and hMPV were significantly more frequent in children and less frequent in 18- to 49-year-olds than in those ≥50 years (*P* = 0·01), fever was more common in hMPV and influenza infections (*P* = 0·008), nasal congestion was more frequent in CoV, HRV, hMPV, influenza and RSV infections (*P* = 0·001), and body mass index was higher among those with influenza (*P* = 0·036).

**Conclusions:**

Using MRT-PCR, a viral etiology was found in three-fifths of patients with medically attended outpatient visits for acute respiratory illness during the influenza season; co-detected viruses were infrequent. Symptoms varied by viral etiology.

## Introduction

Each year hundreds of millions of people are afflicted with respiratory tract infections caused by a wide range of viruses. Most cases of respiratory viral infections are asymptomatic or relatively mild, causing minor illness before the patient fully recovers. Serious illness can develop, especially among certain high-risk groups, leading to hospitalization, worsening of chronic conditions, and even death. In between are cases of moderate to severe respiratory tract infections in which patients seek outpatient medical attention, referred to as medically attended acute respiratory infection (ARI). These infections frequently result in lost productivity in the form of work or school absenteeism or reduced productivity due to attendance at work or school while sick (presenteeism).

The most common viruses associated with respiratory tract infections are human adenovirus (ADNO), human coronavirus (CoV), human metapneumovirus (hMPV), human rhinovirus (HRV), influenza virus (influenza), parainfluenza virus (PIV), and respiratory syncytial virus (RSV). These viral pathogens vary in their pathogenesis, epidemiology, and temporal appearance throughout the year, but typically share a common set of symptoms including cough, fever, and rhinorrhea. Among the many viral ARIs seen in primary care each year, only a portion has been routinely tested to determine the offending organism because (i) rapid testing to differentiate among viruses is not readily available, and (ii) available treatments for most respiratory viruses are chiefly palliative, with the exception being antivirals for treatment of influenza.

New assay methods using multiplex reverse transcriptase polymerase chain reaction (MRT-PCR) are becoming available that allow for relatively rapid detection of multiple virus types. During the 2011–2012 influenza season, the multicenter U.S. Influenza Vaccine Effectiveness (Flu VE) Network conducted a study designed to determine the effectiveness of the season's influenza vaccine. That study used singleplex RT-PCR (SRT-PCR) to detect influenza virus. The University of Pittsburgh site of the Flu VE Network also used MRT-PCR. The purposes of this study were to (i) compare the agreement between SRT-PCR and MRT-PCR for influenza virus detection; (ii) examine the distribution of viruses associated with ARI visits during January through April 2012 in Allegheny County, Pennsylvania, using MRT-PCR; and (iii) compare personal characteristics and symptoms among those infected with various viruses. This study is among the first to examine a wide range of respiratory viral infections, including four CoVs and four PIVs, resulting in outpatient primary care visits among individuals across a broad age spectrum.

## Methods

### Participants

Participants were individuals enrolled in the University of Pittsburgh site of the Flu VE study funded by the Centers for Disease Control and Prevention (CDC) and described previously.^1^ The purpose of the parent study was to determine the effectiveness of seasonal influenza vaccine using a test negative case–control study design,^2^,^3^ where the proportion vaccinated among those who test positive for influenza is compared with the proportion vaccinated among those who test negative. Eligibility criteria included age ≥6 months as of 9/1/2011 and presentation at one of the participating primary care centers for treatment of an upper respiratory illness of ≤7 days duration, with cough or fever, and not taking an influenza antiviral (oseltamivir or zanamivir) before the medical visit. Emergency department visits were not included. Eligible individuals provided written informed consent, completed an enrollment survey, and were swabbed for nasal and oropharyngeal samples. Influenza vaccination status was combined from electronic medical record (EMR) data and self-report. Influenza activity did not increase appreciably over baseline levels as determined by local surveillance systems until mid-January 2012 in the Pittsburgh region; hence, study enrollment began in January and ended in April 2012. This prospective study was approved by the University of Pittsburgh Institutional Review Board.

### Specimen collection

All patients except infants (<2 years) were sampled by two polyester swabs (Remel), one each on the nasal and oropharyngeal mucosa; infants were sampled by nasal swabs only. The swabs were combined in the same cryovial and couriered to the UPMC Clinical Virology Laboratory. The specimens were stored in a lysis buffer and aliquoted for nucleic acid isolation and detection of influenza virus using CDC's SRT-PCR test and a MRT-PCR test using the eSensor XT-8 instrument and respiratory viral panel from GenMark Diagnostics. Funding was available to analyze 662 of the 817 specimens from the parent study with the MRT-PCR. All specimens positive for influenza by SRT-PCR were selected to allow comparison with the MRT-PCR assay and to identify co-infections with influenza; 45 of the 49 SRT-PCR influenza-positive specimens had sufficient sample for MRT-PCR testing. In addition, 617 influenza-negative specimens with sufficient sample were selected for a total of 662 ARI specimens.

### Nucleic acid extraction

Isolation of viral nucleic acid from control material and patient specimens was performed using an EasyMag automated extractor (bioMerieux, Durham, NC, USA) as previously described for the SRT-PCR assay.^4^

### Real-time reverse transcriptase PCR

Previously published, virus-specific primer and probe nucleotide sequences were used for detection of influenza A and B virus RNA.^4^ Detection was performed using the ABI 7500 Real-Time PCR Instrument (Applied Biosystems, Foster City, CA, USA).

### Multiplex PCR

The eSensor RVP multiplex PCR assay (GenMark Diagnostics, Carlsbad, CA, USA) used in our study is currently approved for clinical use in Europe. It has the same methodological characteristics but a broader range of viral analytes than the US FDA approved version. Nucleic acids were extracted as for the SRT-PCR assay, with the addition of 10 μl of bacteriophage MS2 internal control (included in the eSensor RVP kit) to each specimen immediately prior to extraction. Specimens were tested by the eSensor XT-8 instrument and respiratory viral panel according to the manufacturer's instructions and published protocols.^5^ This panel includes adenovirus (ADNO) groups B, C, and E; coronaviruses (CoV) 229E, HKU1, OC43, and NL63; seasonal influenza A virus (including H1N1 and H3N2 subtype determination); influenza B virus; hMPV, PIV types 1, 2, 3, and 4; RSV types A and B; and HRV.

### Demographic and other variables

Participants completed surveys at enrollment from which age, race, personal and household smoking status, household composition, asthma diagnosis, exercise, influenza vaccination status, symptoms of ARI, self-reported overall health before ARI, subjective social status, and self-reported health on day of enrollment were determined. Body mass index (BMI) was calculated from self-reported height and weight.

### Statistical analyses

To reduce the number of small cell sizes, we combined similar viruses (e.g., CoV OC43, HKU1, NL63, and 229E were combined as CoV). Only single viruses or virus groups that were detected in more than 20 samples were used in the analyses, with the final six groups being: no virus detected, HRV, CoV, RSV, hMPV, and influenza virus type A. The measurement of agreement between the singleplex and multiplex influenza assays was determined using Cohen's kappa statistic. Descriptive statistics are presented as means and standard deviations for continuous variables and percentages for discrete variables. Participants were divided into three age groups – children (6 months–17 years), young adults (18–49 years), and older adults (≥50 years). Bivariate multinomial regression models were used to assess the association of patient characteristics with the results of the MRT-PCRs because the probability distribution of the outcome variable consisted of more than two groups. The dependent variable is the virus group, and the independent variables are the participants' personal characteristics. To determine the independent effect, multinomial regression models were fit for each variable controlling for age and self-reported health status, with no virus detected as the reference group. For discrete characteristics with more than two levels that were statistically significant in the adjusted analysis, pairwise multinomial regression models were fit to assess the association of each level; Bonferroni corrections were used to adjust for multiple comparisons. Because the overall effect of age group was significant (*P* = 0·01) in some analyses, pairwise comparisons between the different age groups were conducted to determine which pair of age groups had an effect on the dependent variable. Analyses were conducted using SAS version 9.2 (SAS Institute, Inc., Cary NC, USA).

## Results

### Concordance between SRT-PCR and MRT-PCR for influenza

To ensure the comparability of the SRT-PCR to the MRT-PCR for influenza outcomes, agreement analyses were conducted. Because of the few influenza B cases (*n* = 3) identified, both influenza A and B strains are reported together. There was significant agreement on influenza results between assays (κ = 0·83; chi-square *P* < 0·001, 95% confidence limits = 0·75, 0·92). The sensitivity was 91·1 and specificity was 98·2 when SRT-PCR was treated as the gold standard. Of the 45 singleplex influenza positives, 41 (91%) were multiplex influenza positive (38 influenza A and 3 influenza B), 1 (2%) was multiplex negative for any virus, and 3 (7%) were multiplex positive for non-influenza viruses (HRV, mHMPV, and RSV). The cycle threshold (cT) value for this MRT-PCR influenza-negative but SRT-PCR influenza-positive sample was 29·5; for the other three MRT-PCR negatives for influenza but positive for other viruses, the cT values were 26·1, 29·6, and 25·3. For comparison purposes, the mean cT value for 38 MRT-PCR influenza A positives was 28·9 ± 3·6 (standard deviation). The multiplex assay identified 11 influenza positives not detected by singleplex RT-PCR (7 influenza only, 2 co-detected with RSV, and 1 each co-detected with mHMPV and CoV). The discordant influenza positives were distributed nearly equally among age groups: 3 (27%) for young adults, and 4 (36%) each for children and older adults. Concordance for age groups was high (κ = 0·81–0·87).

### Distribution of viruses and co-infections

Among 662 ARI patients, 349 (52·9%) tested positive for single virus infections, 46 (6·8%) tested positive for more than one virus (i.e., co-detections), and 267 (40·3%) were negative for all tested viruses. The distribution of single virus infections and co-detections are shown in Table1, with the numbers for co-detections representing viruses detected, not numbers of patients. The viruses most frequently co-detected were HRV and CoV (23 occurrences each), followed by influenza (21), ADNO (14), RSV (12), hMPV (9), and PIV (1). The percentages of single, multiple, and no viruses detected varied by age. For children, the percentages of single virus, multiple viruses, and no virus detected were 59%, 12%, and 29%, respectively, whereas for young adults, the percentages were 50%, 3%, and 47%, and for older adults, the percentages were 49%, 5%, and 46%, respectively (*P* < 0·001, data not shown).

**Table 1:** Distribution of viruses from MRT-PCRs among 662 outpatients with medically attended acute respiratory infections

Virus	All samples (*N* = 662) tested by multiplex RT-PCR,* *n* (%)
Singly occurring viruses by type	
Adenoviruses	20 (3·0)
Coronaviruses	69 (10·4)
Influenza viruses	39 (6·0)
hMPV	55 (8·3)
HRV	102 (15·4)
PIV	8 (1·2)
RSV	56 (8·4)
Single viruses total	349 (52·7)
Co-detections	46 (6·9)
No viruses	267 (40·3)

Virus	Co-detected viruses	n**
Co-detected viruses		
ADNO	Dual – CoV, HRV, RSV, Influenza Triple – CoV/RSV, HRV/Influenza	14
CoV	Dual – ADNO, HRV, Influenza, hMPV, RSV Triple – HRV/PIV, HRV/mHMPV	23
HRV	Dual – ADNO, CoV, Influenza, RSV Triple – CoV/PIV	23
Influenza	Dual – ADNO, CoV, HRV, hMPV, RSV Triple – ADNO/HRV	21
hMPV	Dual - CoV, HRV, Influenza, RSV Triple – CoV/HRV	9
PIV	Triple – CoV/HRV	1
RSV	Dual – ADNO, CoV, HRV, Influenza, hMPV Triple – ADNO/CoV, HRV/Influenza	12

CDC, Centers for Disease Control and Prevention method; ADNO, adenovirus; CoV, coronavirus; hMPV, human metapneumovirus; HRV, human rhinovirus; RSV, respiratory syncytial virus.

* MRT-PCR = multiplex reverse transcriptase polymerase chain reaction.

** Represents occurrences of viruses, not individual participants.

**Figure 1:**
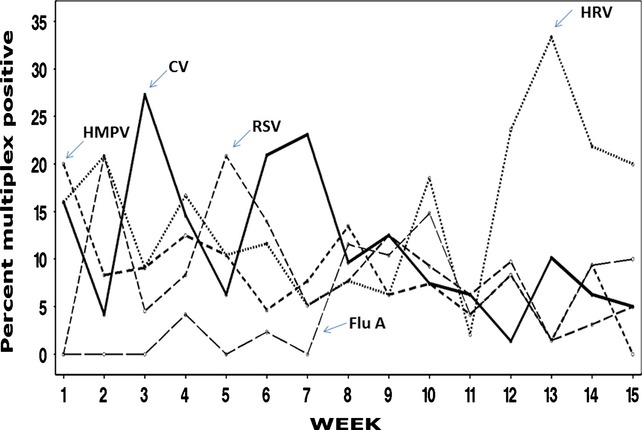
Temporal epidemiology of respiratory viruses by week during 2011–2012.

Among those sampled over 15 weeks, the distributions of viral infections varied by week (*P* < 0·001) and are shown in the Figure1. Overall, CoV infections were readily apparent during late January through early March, RSV was active in January and February, HRV infections increased in April, hMPV infections varied over the time period, and influenza infections peaked in March, during a late and historically light season.

### Relationship of viruses to demographic characteristics and symptoms

Table2 shows the demographic and illness characteristics of patients in five virus groups not including co-infections and those with no virus detected. Mean age, self-rated health status, and BMI all differed significantly across the virus categories, and self-report of cough, fever, sore throat, and nasal congestion at enrollment were all significantly more frequent among those with viral infections than those with no virus detected.

**Table 2:** Relationship of medically attended acute respiratory infections patient characteristics and symptoms to viral etiology in bivariate analyses

Variable	*N*	Virus type						*P* value

		CoV (*n* = 69)	hMPV (*n* = 55)	HRV (*n* = 102)	Influenza (*n* = 36)	RSV (*n* = 56)	No Virus (*n* = 267)	
Categorical demographic variables, row percentages, sum to 100%								
Race, (%)								
White	441	10·4	9·8	19·1	5·4	9·5	45·8	0·21
Other	144	16·0	8·3	12·5	8·3	9·7	45·1	
Age group, (%)								
Children (6 months–17 years)	196	11·2	13·8	17·9	7·6	14·3	35·2	**0·05**
Young adults (18–49 years)	247	14·2	5·3	19·0	4·1	6·5	51·0	
Older adults (≥50 years)	142	8·5	10·6	14·1	7·8	8·5	50·7	
Household members ≥18 years, *n* (%)								
1	135	8·9	8·9	19·3	8·9	11·1	43·0	0·64
2	321	12·5	9·4	17·5	4·4	10·0	46·4	
3	84	11·9	8·3	14·3	8·3	7·1	50·0	
≥4	39	17·9	12·8	17·9	5·1	7·7	38·5	
Household members <18 years, *n* (%)								
0	232	12·1	9·1	16·4	4·7	8·2	49·6	0·49
1	126	11·1	7·1	17·5	6·4	16·7	41·3	
2	120	12·5	10·0	22·5	5·0	9·2	40·8	
≥3	101	10·9	11·9	13·9	10·9	4·9	47·5	
Subjective social status, range, 1 = low to 9 = high (%)								
1–4	95	9·5	8·4	14·7	9·5	8·4	49·5	0·88
5	165	11·5	9·7	17·6	4·2	9·1	47·9	
6	117	17·9	5·1	19·7	4·3	11·1	41·9	
7–9	176	9·7	13·1	17·1	7·9	7·4	44·9	
Health indicators								
Smoker,%	87	13·8	4·6	19·5	4·6	4·6	52·9	0·77
Non-smoker,%	302	11·6	7·6	16·9	6·0	7·6	50·3	
Household smoking,%	123	14·6	6·5	20·3	4·1	8·9	45·5	0·52
No household smoking,%	453	11·3	10·2	16·6	6·8	9·5	45·7	
Asthma diagnosis,%	153	15·0	6·5	17·6	5·2	9·1	46·4	0·60
No asthma diagnosis,%	416	10·8	10·1	17·6	6·7	9·9	45·0	
Received 2011–12 influenza vaccine,%	384	11·2	8·9	17·2	6	12	44·8	0·20
Did not receive 2011–12 influenza vaccine,%	201	12·9	10·5	17·9	6·5	5	47·3	
Exercise < once per month,%	93	10·8	6·5	20·4	6·5	8·6	47·3	0·53*
Exercise > once per month,%	1	0	0	100	0	0	0	
Self-reported health status (%)								
Fair/poor	62	14·5	1·6	21·0	1·6	6·5	54·8	**<0·01**
Good	171	14·6	5·3	21·1	5·3	9·4	44·4	
Very good	221	9·1	10·9	13·1	8·6	9·5	48·9	
Excellent	129	11·6	15·5	18·6	5·4	11·6	37·2	
Symptoms of ARI								
Cough,%	524	12·2	10·1	17·9	6·9	10·1	42·8	<0·01*
No cough,%	61	8·2	3·3	13·1	0	4·9	70·5	
Fatigue,%	453	10·8	9·1	17·2	6·4	9·7	46·8	0·74
No fatigue,%	132	15·2	10·6	18·2	5·3	9·1	41·7	
Fever,%	392	10·5	11	17·4	8·4	10·5	42·4	**<0·01**
No fever,%	193	14·5	6·2	17·6	1·6	7·8	52·3	
Wheezing,%	211	12·8	10·4	18	6·6	11·4	40·8	0·60
No wheezing,%	374	11·2	8·8	17·1	5·9	8·6	48·4	
Sore throat,%	389	11·6	6·9	16·7	5·9	9	49·9	**0·03**
No sore throat,%	196	12·2	14·3	18·9	6·6	10·7	37·2	
Nasal congestion,%	475	13·5	9·5	19·2	6·5	9·9	41·5	**<0·01**
No nasal congestion,%	110	4·6	9·1	10	4·6	8·2	63·6	
Shortness of breath,%	236	12·7	8·5	17·4	6·4	10·6	44·5	0·94
No shortness of breath,%	349	11·2	10	17·5	6	8·9	46·4	

Continuous demographic variables							
Age, years, mean (SD)	29·9 (20)	26·1 (25·3)	28·9 (20·8)	30·1 (24)	26·0 (24·5)	34·2 (20·6)	**0·03**
Body mass index, mean (SD)	28·9 (10·3)	23·7 (7·2)	26·4 (7·8)	29·6 (14·4)	25·5 (8·2)	27·2 (7·8)	**0·01**
Number of times exercise per month, mean (SD)	12·9 (8·6)	14·6 (14·6)	13·5 (8·0)	9·9 (7·5)	15·3 (11·7)	15·1 (15·1)	0·42
Rating of health at enrollment, range, 1 = low to 100 = high), mean (SD)	58·9 (17)	61·6 (19·9)	60·4 (19·9)	51·7 (21·1)	55·4 (22·1)	58·9 (19·1)	0·23
Days from illness onset to enrollment, mean (SD)	3·3 (1·5)	3·5 (1·6)	3·5 (1·6)	3·3 (1·5)	3·5 (1·8)	3·4 (1·8)	0·91

CoV, coronavirus; hMPV, human metapneumovirus; HRV, human rhinovirus; RSV, respiratory syncytial virus; ARI, acute respiratory infection.

*P* values are obtained from the Wald chi-square statistics using multinomial logit regression modeling.

* Based on Fisher's exact test.

Bold indicates significant P values.

In adjusted multinomial regression analyses, type of virus infection significantly differed by age group, BMI, and presence of fever and nasal congestion (Table3). Children were more likely to have hMPV and RSV detected than older adults, while young adults were less likely to have hMPV, RSV, and influenza viruses detected than were older adults. Those presenting with fever were more likely to have hMPV or influenza than other viral infections, and those presenting with nasal congestion were more likely to have CoV and HRV than other viral infections. Individuals with influenza and CoV had higher BMIs than those with other types of infections. Although overall influenza vaccination status did not differ across viral etiologies, those who were vaccinated against influenza were more likely to have RSV virus detected, compared with those who were not vaccinated. The proportion of those reporting cough, wheezing, and sore throat did not differ significantly across viral infections, but cough was more likely to be reported with hMPV, HRV, and RSV infection, wheezing more likely with hMPV and RSV infection, while sore throat was less likely to be reported with hMPV infection.

**Table 3:** Relationship of demographic characteristics and symptoms of patients presenting with medically attended acute respiratory infections to viral etiology in multinomial regression analyses, controlling for age and self-reported health status

	Virus type					
	
	CoV (*n* = 69)	hMPV (*n* = 55)	HRV (*n* = 102)	Influenza (*n* = 36)	RSV (*n* = 56)	Adjusted *P* value
Categorical variables, odds ratio (95% confidence interval)						
Race, white versus other	0·84 (0·63–1·12)	1·12 (0·77–1·62)	1·28 (0·95–1·72)	0·80 (0·54–1·17)	1·03 (0·73–1·44)	0·185
Age group*						
6 months–17 years versus ≥50 years	1·37 (0·90–2·08)	**1·60 (1·05**–**2·44)**	1·39 (0·98–1·98)	1·44 (0·87–2·38)	**1·91 (1·26**–**2·90)**	**0·010**
18–49 years versus ≥50 years	1·12 (0·78–1·60)	**0·53 (0·33**–**0·85)**	0·99 (0·73–1·35)	0·60 (0·36–1·00)	**0·63 (0·41**–**0·97)**	
Household members ≥18 years, (*n*)*						
1 versus ≥4	0·73 (0·42–1·28)	0·98 (0·54–1·75)	1·16 (0·74–1·82)	1·46 (0·77–2·79)	1·32 (0·74–2·35)	0·815
2 versus ≥4	0·96 (0·64–1·46)	0·88 (0·55–1·42)	0·97 (0·67–1·42)	0·64 (0·35–1·18)	1·06 (0·65–1·74)	
3 versus ≥4	0·86 (0·47–1·55)	0·73 (0·37–1·46)	0·74 (0·43–1·28)	1·13 (0·54–2·36)	0·70 (0·34–1·46)	
Household members <18 years, (*n*)*						
0 versus ≥3	0·92 (0·61–1·39)	1·00 (0·63–1·60)	0·85 (0·59–1·22)	0·71 (0·40–1·27)	0·87 (0·54–1·41)	0·210
1 versus ≥3	1·04 (0·63–1·72)	0·79 (0·43–1·43)	1·09 (0·71–1·68)	1·06 (0·56–2·01)	**2·01 (1·23**–**3·28)**	
2 versus ≥3	1·20 (0·72–1·97)	1·03 (0·60–1·80)	1·43 (0·95–2·17)	0·81 (0·40–1·64)	1·08 (0·61–1·92)	
Subjective social status (range, 1 = low to 9 = high)						
1 versus ≥7	0·71 (0·39–1·28)	1·13 (0·59–2·16)	0·75 (0·45–1·23)	1·59 (0·83–3·06)	0·95 (0·50–1·80)	0·438
5 versus ≥7	0·93 (0·59–1·47)	1·14 (0·68–1·90)	0·97 (0·66–1·43)	0·68 (0·35–1·33)	1·00 (0·60–1·66)	
6 versus ≥7	**1·68 (1·06**–**2·67)**	0·61 (0·31–1·23)	1·26 (0·82–1·94)	0·75 (0·35–1·59)	1·33 (0·77–2·28)	
Health indicators						
Smoker versus non-smoker	0·94 (0·43–2·03)	0·90 (0·28–2·89)	1·03 (0·53–2·02)	0·93 (0·29–3·02)	0·60 (0·19–1·90)	0·974
Household smoking versus no household smoking	1·16 (0·62–2·20)	0·78 (0·34–1·79)	1·15 (0·66–2·02)	0·64 (0·23–1·76)	0·92 (0·43–1·95)	0·860
Asthma diagnosis versus no asthma diagnosis	1·28 (0·72–2·30)	0·75 (0·35–1·60)	0·94 (0·55–1·59)	0·81 (0·35–1·88)	0·94 (0·48–1·86)	0·863
Received 2011–12 influenza vaccine	0·89 (0·51–1·55)	0·96 (0·52–1·78)	1·00 (0·62–1·61)	0·99 (0·48–2·04)	**2·53 (1·22**–**5·26)**	0·216
Self-rated health status;** reference = Excellent						
Fair/poor	1·09 (0·60–2·00)	0·23 (0·05–1·06)	1·06 (0·63–1·79)	0·31 (0·07–1·45)	0·69 (0·30–1·57)	0·054
Good	1.30 (0·84–2·00)	0·91 (0·43–1·91)	1·26 (0·86–1·84)	1·24 (0·58–2·65)	1·16 (0·69–1·95)	
Very good	0·67 (0·43–1·05)	1·62 (0·86–3·07)	**0·66 (0·45**–**0·97)**	1·79 (0·91–3·50)	0·94 (0·58–1·52)	
Symptoms of ARI						
Cough***	2·5 (0·95–6·59)	**10·6 (1·41**–**79·1)**	**2·29 (1·04**–**5·08)**	–	3·46 (1·03–11·6)	–†
Fatigue	0·69 (0·37–1·27)	0·94 (0·46–1·91)	0·92 (0·53–1·61)	1·16 (0·48–2·83)	1·14 (0·55–2·35)	0·838
Fever	0·85 (0·50–1·48)	**2·26 (1·10**–**4·64)**	1·17 (0·72–1·90)	**6·62 (1·97**–**22·2)**	1·53 (0·80–2·92)	**0·008**
Nasal congestion	**4·66 (1·80**–**12·1)**	1·82 (0·84–3·97)	**3·02 (1·52**–**5·98)**	2·24 (0·84–6·00)	1·93 (0·90–4·18)	**0·001**
Shortness of breath	1·25 (0·70–2·20)	1·35 (0·71–2·56)	1·11 (0·68–1·83)	1·37 (0·65–2·88)	1·66 (0·89–3·09)	0·650
Sore throat	0·76 (0·43–1·35)	**0·40 (0·22**–**0·74)**	0·71 (0·43–1·16)	0·69 (0·33–1·45)	0·71 (0·38–1·31)	0·102
Wheezing	1·44 (0·81–2·55)	**2·07 (1·09**–**3·94)**	1·36 (0·83–2·23)	1·59 (0·75–3·37)	**2·07 (1·11**–**3·87)**	0·104
Continuous Variables odds ratio (95% confidence interval)**						
Age* (units = 5)	0·95 (0·89–1·01)	0·95 (0·88–1·02)	**0·94 (0·88**–**0·99)**	0·97 (0·89–1·06)	**0·92 (0·86**–**0·99)**	0·104
BMI (units = 5)	**1·22 (1·02**–**1·45)**	0·84 (0·65–1·10)	1·02 (0·86–1·20)	**1·32 (1·06**–**1·64)**	0·98 (0·78–1·25)	**0·036**
Rating of health at enrollment (units = 5)	0·99 (0·92–1·07)	0·99 (0·91–1·08)	1·02 (0·95–1·08)	**0·89 (0·81**–**0·98)**	0·93 (0·86–1·00)	0·066
Days from illness onset to enrollment (*n*)	0·98 (0·84–1·15)	1·07 (0·90–1·27)	1·07 (0·93–1·22)	0·99 (0·81–1·22)	1·06 (0·89–1·26)	0·874
Days of exercise per month (*n*; units = 5)	0·91 (0·74–1·10)	1·00 (0·81–1·23)	0·94 (0·80–1·10)	**0·68 (0·47**–**0·98)**	1·01 (0·87–1·18)	0·389

ARI, medically attended acute respiratory infection; CoV, coronavirus; hMPV, human metapneumovirus; HRV, human rhinovirus; RSV, respiratory syncytial virus.

Multinomial regression modeling was used; virus group = dependent variable; independent variables are shown in Column 1. Pairwise comparisons for age groups were conducted; *P* ≥ .057.

* Adjusted for self-reported health status only.

** Adjusted for age group only.

*** Cough was an inclusion criterion.

† Model does not converge.

Those with more than one virus detected were more likely to be young, to have been vaccinated against influenza, and to have a lower BMI than those presenting with a single viral infection (Table4).

**Table 4:** Demographic and other characteristics of patients presenting with co-infections (*n* = 46) versus other single viral infections (*n* = 349)

Variable	Co-infection (*n* = 46)	*P* value*	Unadjusted odds ratio (95%CI)
Categorical variables			
Race, white	12·3	0·499	1·30 (0·60–2·81)
Race, other	9·7		Reference
Age group**		**0·014**	
6 months–17 years	17·4		1·99 (0·87–4·58)
18–49 years	6·3		0·63 (0·24–1·71)
≥50 years	9·5		Reference
Household members ≥18 years, (*n*)**		0·791	
1	13·5		2·11 (0·45–9·97)
2	11·3		1·72 (0·38–7·71)
3	13·0		2·01 (0·39–10·4)
≥4	6·9		Reference
Household members <18 years, (*n*)**		0·651	
0	8·6		0·63 (0·25–1·58)
1	12·0		0·91 (0·35–2·32)
2	13·5		1·04 (0·41–2·63)
≥3	13·0		Reference
Subjective social status (range, 1 = low to 9 = high)		0·211	
1–4	12·7		0·97 (0·39–2·41)
5	6·1		0·44 (0·16–1·16)
6	16·1		1·28 (0·9–2·79)
≥7	13·0		Reference
Health indicators			
Smoker	5·7	0·494	0·64 (0·18–2·30)
Non-smoker	8·6		Reference
Household smoking	7·2	0·149	0·52 (0·21–1·27)
No household smoking	13·1		Reference
Asthma diagnosis	10·7	0·643	0·84 (0·41–1·73)
No asthma diagnosis	12·4		Reference
Received 2011–12 influenza vaccine	13·8	**0·037**	2·33 (1·05–5·17)
Did not receive 2011–12 influenza vaccine	6·4		Reference
Self-rated health status†		0·838	
Fair/poor	8·8		0·60 (0·16–2·21)
Good	11·0		0·76 (0·34–1·70)
Very good	11·4		0·79 (0·37–1·69)
Excellent	14·0		Reference
Symptoms of ARI			
Cough	11·9	0·519	1·63 (0·37–7·11)
No cough	7·7		Reference
Fatigue	12·3	0·499	1·30 (0·60–2·81)
No fatigue	9·7		Reference
Fever	11·4	0·771	0·91 (0·46–1·77)
No fever	12·4		Reference
Nasal congestion	10·8	0·192	0·60 (0·28–1·29)
No nasal congestion	16·7		Reference
Shortness of breath	8·3	0·094	0·56 (0·28–1·10)
No shortness of breath	13·9		Reference
Sore throat	9·9	0·168	0·65 (0·35–1·20)
No sore throat	14·5		Reference
Wheezing	11·2	0·821	0·93 (0·49–1·76)
No wheezing	11·9		Reference
Continuous variables			
Age** (units = 5)	19·7 (22·8)	**0·015**	0·91 (0·83–0·98)
BMI (units = 2)	22·8 (7·1)	**0·010**	0·89 (0·81–0·97)
Rating of health at enrollment (units = 5)	61·5 (18·6)	0·249	1·05 (0·97–1·13)
Days from illness onset to enrollment (*n*)	3·3 (1·8)	0·825	0·98 (0·81–1·18)
Days of exercise per month (units = 5)	8·9 (6·4)	0·084	0·70 (0·47–1·05)

CI, confidence interval.

For continuous variables (e.g., Age), the OR is relative to an increase in the measurement by the number of units indicated.

* *P* value for Wald chi-square tests and unadjusted regression analyses.

** Adjusted for self-reported health status only.

† Adjusted for age only

## Discussion

In a sample of 662 outpatients with ARI of 7 or fewer days duration during January–April 2012, one or more viruses was detected in 395 nasal/oropharyngeal samples using MRT-PCR assays capable of detecting 18 virus types. Viruses detected included ADNO, CoV, HRV, hMPV, influenza, RSV, and PIV. We found a high correlation between the MRT-PCR and CDC's SRT-PCR test for detection of influenza virus (κ = 0·83, 95% CI = 0·81–0·87) which was slightly lower than one report (κ = 0·96),^5^ but similar to another (κ = 0·84),^6^ thus expanding influenza assay options.

Co-detections were infrequent (6·9%); rates ranged from 12% in children to 3% in young adults to 5% in older adults, comparable to 10·5% co-infection reported among adults and children presenting to emergency departments^7^ and 4·9% reported in pediatric patients with respiratory symptoms.^6^ In contrast, among children attending day care, 46% of respiratory illnesses were attributed to co-infections.^8^ Our sample sizes were insufficient to analyze co-detections by age group, but age-group analysis warrants further study.

Co-detected viruses may represent either two clinically significant infections or one infection significant enough to lead to a medical visit and a second clinically insignificant/asymptomatic infection. Co-infections with certain viruses may increase the severity of some respiratory infections with the possibility that specific pairings of viruses may determine symptoms and duration of illness.^9^ Recent research suggests that certain co-occurrences depend upon the co-infecting viruses. For example, Greer et al. reported a negative association between HRV and co-infections with ADNO, CoV, hMPV, RSV, and influenza, among others,^10^ while Martin et al. reported a similar negative relationship between HRV and RSV.^8^ Conversely, Tanner et al. reported a significant positive relationship between HRV and RSV, but reported negative associations between influenza A(H1N1)pdm09 and hMPV and RSV.^11^ In our study, HRV was the most frequently occurring single virus and was also the most frequent co-detected virus.

The associations between viral infections and demographic and symptom variables were not unexpected. For instance, hMPV, RSV, and influenza are more common among young children and older adults^12^ than among younger adults. Fever is a common symptom for both hMPV and influenza.^13^ We found higher BMI to be associated with influenza infection leading to ARI. Previous research has found changes in certain immune functions, and less robust immune response to vaccines is related to increased body weight,^14^ and obese patients frequently experience worse outcomes related to infectious diseases than their thinner counterparts.^15^ Talbot et al. reported a positive association between obesity and seroconversion against the H3N2 component of influenza vaccine, but not against the H1N1 or B strains,^16^ but found no relationship between obesity and seroprotection against any influenza strain tested. The specific mechanism for higher influenza disease rates among those with higher BMIs is unclear.

We found several demographic/household differences between those with co-detected viruses compared with single virus infections, including, being younger (expected), having a lower BMI, and more likely to have been vaccinated against influenza. This is in contrast to a recent study of children attending day care in which no demographic/household variables were related to co-infections and presence of fever was less likely.^17^ Nonetheless, further studies with larger sample sizes would allow confirmation of the univariate associations we observed, as well as multivariate analysis to rule out potential confounders.

### Strengths and limitations

This study offers data on viral infections associated with outpatient ARI in the US during the winter influenza season, using the eSensor 18 virus panel currently available in Europe. This panel of viruses is an expanded version that includes several viruses not part of the FDA-cleared format, that is, the four CoVs and four PIVs. The test involves bioelectronic detection of viral DNA amplified from nucleic acid extracted from the specimen by a conventional RT-PCR assay.^18^ The assay compares favorably to singleplex RT-PCR in test characteristics, albeit A(H3N2) was the predominant strain in the year tested, yielding little information on the other strains.^5^ Study limitations include coverage of a single season when influenza was notably light in Pittsburgh and when A(H3N2) predominated, and inability to test an inclusive sample of all of the ARI specimens with the MRT-PCR. However, the sample size was sufficient to allow confidence in the relationships between characteristics of ARI cases and the viruses associated with them during this time period. Additionally, the viral panel does not contain bacteria, mycoplasma, and all possible respiratory viruses, and some viruses could occur more frequently in other seasons.

## Conclusions

Using multiplex RT-PCR, three-fifths of medically attended outpatient visits for acute respiratory illness during the winter were associated with a viral etiology. Viruses differed in distribution across age groups, self-reported health status, BMI, presence of fever, and nasal congestion. Co-infection was present in a small proportion of cases, but varied by age, weight, and influenza vaccination status.
